# Attending Icu Physicians And Residents Do Not Agree About The Applicability of Advance Directives

**DOI:** 10.1186/2197-425X-3-S1-A36

**Published:** 2015-10-01

**Authors:** NH Leder, D Schwarzkopf, K Reinhart, C Hartog

**Affiliations:** University Hospital Jena, Jena, Germany

## Introduction

In Germany, advance directives (AD) are written documents explaining what medical treatment the individual would prefer in the future, should that individual lack mental capacity. AD are legally binding. When a patient has lost mental capacity, the treating physician judges whether the AD is applicable or not. The AD is applicable if the patient's present condition matches the clinical scenario the patient specified in writing (in the following termed “applicability statement”). Common applicability statements are “in the event of a terminal illness” or “if I have irreversible organ failure”. It is unknown how ICU physicians judge the applicability of a given ADs.

## Objectives

To compare the judgment of attending, i.e. experienced intensivists and the judgment of residents in critical care training.

## Methods

Prospective observational patient-based study in 4 multidisciplinary ICUs of a German university hospital. Patients were included if stayed >48 hours, were mentally incompetent and had a written AD. The structured interviews took place on the day of patient inclusion. Treating physicians were provided with verbatim copies of the relevant statements and asked whether these matched the medical situation. The study was IRB approved.

## Results

Fifty ICU patients with ADs were included; 33 (66%) patients were mechanically ventilated. 78% of AD contained pre-printed applicability statements. 84% of ADs contained 3 or more applicability statements. All ADs, had they been applicable, refused life-supporting measures. 39 ICU physicians participated (14 consultants and 25 residents). Consultants had more experience with AD than residents (p = 0.003). Attendings judged overall 43 AD, residents judged 46 AD. a direct comparison of the judgment of both groups was possible in 39 cases. Physicians judged that 17 of 39 AD were applicable at the time of the interview, but attendings' and residents' judgment agreed in only 6 cases. Cohens Kappa regarding the agreement of judgments was 0.13 [95% confidence intervall -0.09, 0.35], i.e. there was no significant agreement between the groups. AD contained overall 153 single applicability statements, of which 25 were judged as applicable, i.e. that the current clinical condition matched the patient's written statement. But only 5 statements were judged applicable by both groups (Cohens Kappa 0.17 [0.03, 0.31]), indicating no significant agreement.

## Conclusions

Experienced and inexperienced intensivists vary considerably in their interpretation of an AD in the ICU. Given that the number of patients with an AD is growing, residency training should include structured education on how to interpret an AD and respect the patient's preference even in the challenging setting of an ICU.

## Grant Acknowledgment

The study was partly funded by the German Ministry for Education and Research and the Interdisciplinary Center for Clinical Research of the Jena University Hospital.

